# Familial assimilation in transmission of raw-freshwater fish-eating practice leading to clonorchiasis

**DOI:** 10.1371/journal.pntd.0008263

**Published:** 2020-04-30

**Authors:** Men-Bao Qian, Zhi-Hua Jiang, Chang-Hai Zhou, Tao Ge, Xin Wang, Xiao-Nong Zhou

**Affiliations:** 1 National Institute of Parasitic Diseases, Chinese Center for Disease Control and Prevention, Shanghai, China; 2 Chinese Center for Tropical Diseases Research, Shanghai, China; 3 Key Laboratory of Parasite and Vector Biology, Ministry of Health, Shanghai, China; 4 National Center for International Research on Tropical Diseases, Ministry of Science and Technology, Shanghai, China; 5 WHO Collaborating Center for Tropical Diseases, Shanghai, China; 6 Guangxi Center for Disease Control and Prevention, Nanning, Guangxi, China; 7 Heilongjiang Center for Disease Control and Prevention, Haerbin, Heilongjiang, China; 8 Jilin Center for Disease Control and Prevention, Changchun, Jilin, China; Seoul National University College of Medicine, REPUBLIC OF KOREA

## Abstract

Clonorchiasis is caused by raw-freshwater fish-eating practice and causes high burden in Asia. Transmission mechanism of this behavior hasn’t been illuminated, which hinders the adoption of sustainable control activities. A cross-sectional survey was implemented in students from four endemic provinces in China. Data with 23,222 students aged 9–18 and their parents were eligible. Familial clustering of raw-eating practice, impact of parents’ practice on children, interaction of spouses’ practice was analyzed. Raw-eating practice met β-binomial distribution (*χ*^2^ = 0.8, p>0.05). Clustering coefficient increased by students’ age (*R*^2^ = 0.82, p<0.001) and was higher in those families with boys compared to girls (*t* = 4.1, p<0.01). The proportion of students with raw-eating practice increased yearly by 8.9% in girls and 10.5% in boys. Compared to those without parents’ raw-eating practice, adjusted odds ratio of students’ raw-eating practice was 10.5 (95% confidential intervals (95% CI): 9.4–11.7) in those with fathers’ practice, 33.6 (95% CI: 26.3–42.9) in those with mothers’ practice and 47.1 (95% CI: 42.0–52.8) in those with both parents’ practice. There existed interaction between spouses’ practice (*χ*^2^ = 6713.1, p<0.001) and the impact from husband on his wife was higher than that from wife on her husband. Familial assimilation characterizes the transmission of raw-freshwater fish-eating practice, consisted of vertical intergenerational assimilation from parents to their children and horizontal martial assimilation between spouses. A sustainable strategy against clonorchiasis should interrupt the transmission of raw-freshwater fish-eating practice. Additionally, further studies are expected to explore more information, e.g. the frequency in raw-eating practice and type of raw freshwater fish, infection status of *C*. *sinensis* in participants, as well as direct collection of parents’ eating information from themselves.

## Author summary

Clonorchiasis is caused by the ingestion of raw freshwater fish, which contains the infective larvae of *Clonorchis sinensis*. It is highly endemic in Asia, where about 15 million people are afflicted. To establish sustainable control strategy, the transmission of raw-eating practice needs to be illuminated. In this study, we conducted a survey in school students from four clonorchiasis endemic provinces in China, covering 23,222 students aged 9–18. The characteristics of raw-eating practice, impact of parents’ raw-eating practice on children, interaction of spouses’ practice was explored. It is demonstrated that raw-eating practice presents familial clustering, which is higher in those families with older children and with boys. Raw-eating practice in children is highly influenced by their parents’ raw-eating practice especially when both parents do. Additionally, there exists interaction between spouses’ raw-eating practice. The impact of husband’s raw-eating practice on his wife is higher than that of wife’s raw-eating practice on her husband. Familial assimilation dominates the transmission of raw-freshwater eating-practice, including the assimilation from parents to their children and that between spouses. This finding indicates the adoption of sustainable strategy against clonorchiasis through blocking raw-freshwater fish-eating practice.

## Introduction

Clonorchiasis is caused by eating raw freshwater fish, which harbour the infective larvae (metacercariae) of *Clonorchis sinensis* [1]. The adult worms parasitize in the liver and biliary system and subsequently cause damages there. At the early stage of infection, no or only some mild symptoms are presented, e.g. abdominal pain and diarrhoea [1, 2]. However, long and chronic infection would lead to severe liver and biliary complications including gallstone and cholecystitis [1, 3, 4]. In particular, if untreated, *C*. *sinensis* infection would cause fatal cholangiocarcinoma-the bile duct cancer [5, 6].

Clonorchiasis is predominantly endemic in East Asia, including China, South Korea, northern Vietnam and part of Russia, which is relevant to the deeply rooted raw-freshwater fish-eating dietary practice there [7–10]. Overall, an estimation of 15 million people is afflicted, of which 13 million cases distribute in China [7, 8]. Although clonorchiasis has been reported from two dozen provinces in China, over 90% cases come from four eastern provinces, namely Guangxi and Guangdong in the southeast and Heilongjiang and Jilin in the northeast [11]. The dietary habit of ingesting raw freshwater fish could date back to one century ago there [12]. Following the development of aquaculture, the endemicity is worsen in some areas because of the more accessibility to freshwater fish [13, 14]. The epidemiology of clonorchiasis is characterized by the increasing prevalence and intensity by ages and higher prevalence and intensity in males compared to females [7, 11, 13]. These differences are predominantly attributable to the variations in raw-eating practice other than biological factors as susceptibility [15].

Preventive chemotherapy with praziquantel is currently the mainstream against clonorchiasis and recommended by the World Health Organization, which could control the morbidity effectively in short run [16, 17]. A coverage of 75% by preventive chemotherapy of population at risk by 2020 has been set by the World Health Organization [18]. However, preventive chemotherapy couldn’t prevent re-infection and thus the effectiveness is usually unsustainable [17, 19]. Thus, education is usually advocated to abandon the raw-eating practice [20, 21]. However, raw-freshwater fish-eating habit is deep-rooted in adults, who are highly afflicted by clonorchiasis [7, 11, 13]. Paradoxically, adults usually have more knowledge on clonorchiasis due to wide social activities, which indicates the limited effectiveness of education on them [22]. Thus, it is of value to illuminate the transmission mechanism of raw-freshwater fish-eating practice in population, which would benefit the design of sustainable intervention strategy.

## Materials and methods

### Ethics statement

The study was approved by the ethics committees in the National Institute of Parasitic Diseases, Chinese Center for Disease Control and Prevention (reference no. 20170711).

The objectives, procedures and potential risks of this study were orally explained and informed to the principals of the schools and all participants and informed consent was also obtained.

### Study design and survey organization

**Fig 1:**
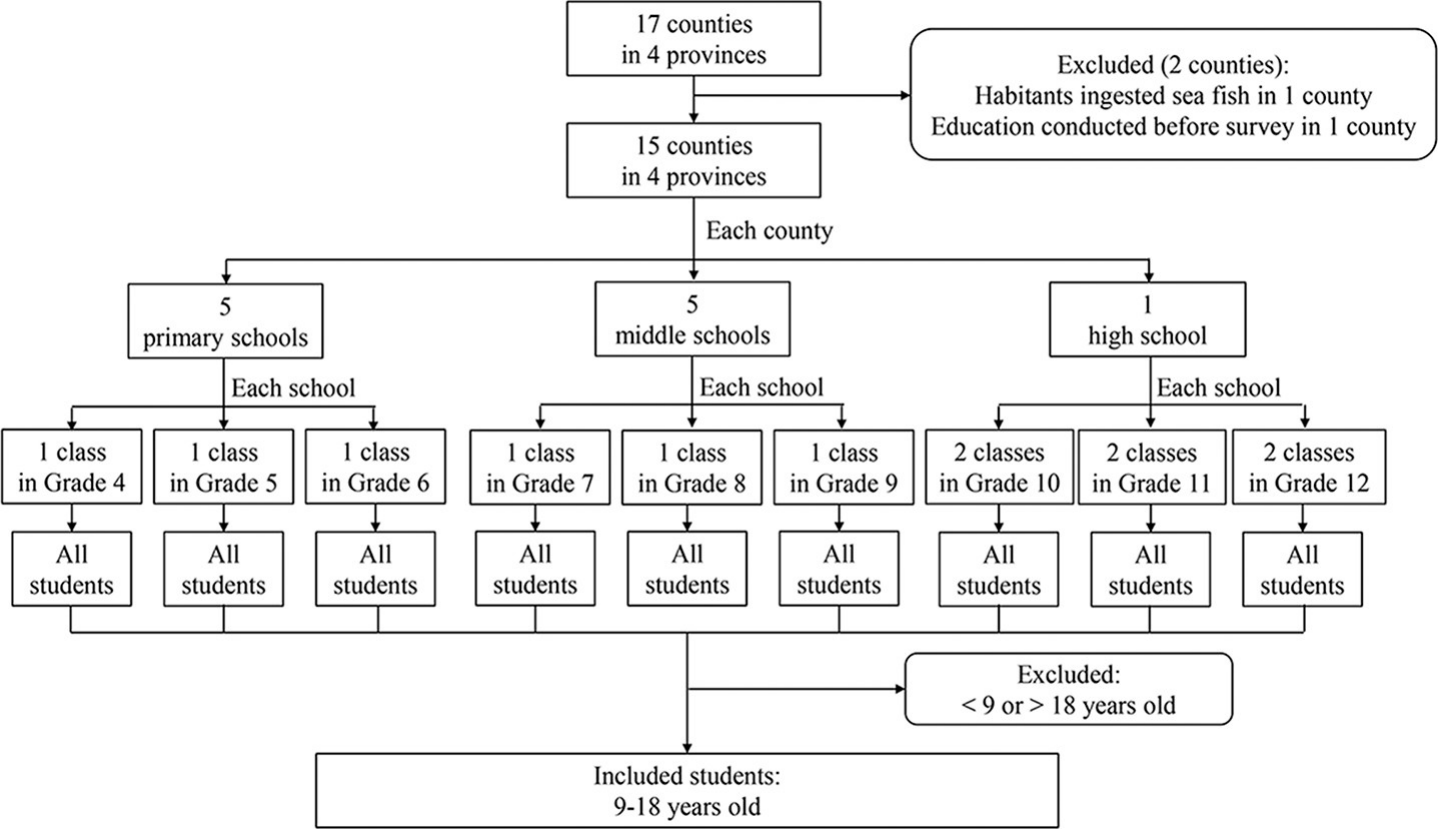
Flow chart of the participants.

From 25 August to 20 December, 2017, surveys were implemented in 17 counties from four major provinces with clonorchiasis in China, including Guangxi and Guangdong in the southeast and Heilongjiang and Jilin in the northeast (**S1 Checklist** and **Fig 1**). In each county, one high school was firstly selected and then two classes in Grade 10, two classes in Grade 11 and two classes in Grade 12 were selected. In each county, five middle schools were firstly selected and then one class in Grade 7, one class in Grade 8 and one class in Grade 9 were selected from each middle school. In each county, five primary schools were firstly selected and then one class in Grade 4, one class in Grade 5 and one class in Grade 6 was selected from each primary school. Overall, in each county, 15 classes from five primary schools, 15 classes from five middle schools and six classes from one high school were enrolled. Then, all students in the classes were eligible in the survey.

### Procedures

A questionnaire was distributed to each student. The staff firstly introduced the questionnaire and then the students filled out the form. The content of the questionnaire included the demography, the knowledge on clonorchiasis, the practice of and belief on raw-freshwater fish-eating. Two items were set on the knowledge. One item focused on the transmission route of clonorchiasis, which included one right answer and other five confusing answers. Another item inquired the harms of clonorchiasis, which included three right answers and three confusing answers. The three right answers involved the damages of clonorchiasis at three different levels, namely the piece of knowledge on early symptoms (abdominal pain and diarrhea), on later complications (gallstone and cholecystitis) and on fatal sequence (carcinogenicity). The belief aimed to inquire the ability of the students not to eat raw freshwater fish in future if required. Students were also asked to report whether they had seen their parents’ practice of eating raw freshwater fish.

### Statistical analysis

Data were analyzed in SPSS for Windows and Microsoft Excel. To avoid the casual selection by the students, only when the right answer was selected and other confusing answers were not selected, then was the knowledge on transmission route judged as known. As to the knowledge on harms, the algorithm below was applied. Firstly, the right answer and wrong one was assigned 1 and -1, respectively. Secondly, the total score was added. Finally, when the total score was over 0 (namely 1, 2 or 3), and one right answer (one of the three pieces of knowledge) was selected, then this piece of knowledge was judged as known. The answers to the belief were polytomous (including yes, no and uncertain), which was transformed into dichotomous through the combination of the answers of no and uncertain into one group, namely no/uncertain. Based on parents’ raw-freshwater fish-eating practice, a family was classified into each of four groups, namely with neither parents’ eating raw freshwater fish, father’s eating, mother’s eating and both parents’ eating.

To detect the familial aggregation in practice, β-binomial distribution and binomial distribution were used [23, 24]. The Clustering coefficient (θ) was then employed in β-binomial distribution to demonstrate the clustering degree. Then, paired Student’s t test was employed to detect the difference between genders and linear equation was used to detected the trend by ages in which determinant coefficient (*R*^2^) was presented. Descriptive analysis was employed to demonstrate the practice and belief by genders and ages and Chi-square test was applied. Linear regression was used to explore the trend of variables by ages of students. Based on the linear regression equations, the changing trends of practice and belief by ages were predicted. Univariate logistic regression analysis and then multivariate one were applied to explore the determinants of students’ practice and belief, in which odds ratio (OR), adjusted OR (aOR) and their 95% confidential intervals (95% CI) were employed. Forward Wald method was used in selection of variables in multivariate logistic regression. The interaction of parents’ practice was analyzed through comparing the percentage of fathers’ (mothers’) eating practice based on mothers’ (fathers’) practice in which the relative risk (RR) was calculated.

## Results

### Profiles of participants

Seventeen counties from four provinces were surveyed. People had the habit of ingesting raw sea fish other than freshwater fish in one county. Health education was carried out immediately before the survey in another county. Thus, data from other 15 counties were included, involving 23,369 students from 75 primary schools, 75 middle schools and 15 high schools. Among them, 11,758 were girls and 11,611 were boys. Ages ranged from 7 to 20 years old, most of which (23,222, 99.4%) between 9 and 18. The number of students aged 7, 8, 19 and 20 was all less than 100, and thus they were excluded in subsequent analysis. Thus, 23,222 students were final included.

Totally, 8,006 students (34.5%) knew transmission route of clonorchiasis, 6,395 (27.5%) knew early symptoms, 3,864 (16.6%) knew complications, and 1,777 (7.7%) knew carcinogenicity. Overall, 3,718 students (16.0%) reported raw-freshwater fish-eating practice of themselves. Most students (19,024, 81.9%) believed that they could do if required not to eat raw freshwater fish in future.

Among 23,222 families, the percentage of fathers with raw-freshwater fish-eating practice was 30.1%, while that of mothers was 14.2%. The composition of the family without parents’ raw-eating practice, with only fathers’ raw-eating practice, with only mothers’ raw-eating practice and with both parents’ raw-eating practice was 68.6%, 17.2%, 1.3% and 12.9%, respectively.

### Familial aggregation in practice

The raw-freshwater fish-eating practice met β-binomial distribution (*χ*^2^ = 0.8, p>0.05) other than binomial distribution (*χ*^2^ = 19553.2, p<0.001) in family. The Clustering coefficient (θ) was 0.87. In those families with girls, θ increased by ages of girls insignificantly (p>0.05). In those families with boys, θ increased by ages of boys significantly (*R*^2^ = 0.87, p<0.001). The *R*^2^ reached 0.82 in combined groups (p<0.001). Overall, θ was higher in those families with boys compared to those with girls (*t* = 4.1, p<0.01).

### Determinants of students’ practice

The percentage of raw-freshwater fish-eating practice in boys (19.8%) was higher than that in girls (12.3%) (*χ*^2^ = 241.2, p<0.001) and this difference was demonstrated significantly in all ages (**S1 Table**). There existed significant linear increasing trends by ages in both genders (**Table 1**) and the corresponding *R*^2^ was 0.90, 0.97 and 0.99 in girls, boys and both (p<0.001 in all). Based on the linear trends, raw-freshwater fish-eating practice increased by 115.4% in girls and 145.9% in boys from 9 to 18. The annual increasing rates were 8.9% and 10.5%, correspondingly.

**Table 1:** Changing trends of practice and belief by ages of students.

Items	Linear regression equations	*R*^2^	p
**Raw-freshwater fish-eating practice**			
**Girls**	Y = 1.0274X-1.2339	0.90	0.000
**Boys**	Y = 1.9114X-5.4084	0.97	0.000
**Both**	Y = 1.4575X-3.2053	0.99	0.000
**Belief on the ability not to eat raw freshwater fish in future**			
**Girls**	Y = -0.6915X+94.7100	0.44	0.036
**Boys**	Y = -1.4572X+96.3760	0.81	0.000
**Both**	Y = -1.0508X+95.2790	0.70	0.003

In multivariate logistic regression, gender, ages, knowledge on early symptoms and parents’ raw-freshwater fish-eating practice were relevant to the students’ raw-eating practice (**Table 2**). The aOR was 2.1 (95% CI: 1.9–2.3) in boys compared to girls, 1.1 (95% CI: 1.1–1.1) following the increase by one year in ages and 1.2 (95% CI: 1.1–1.3) with the knowledge on early symptoms. Compared to those students without parents’ raw-freshwater fish-eating practice, the aOR was 10.5 (95% CI: 9.4–11.7) in those with fathers’ practice, 33.6 (95% CI: 26.3–42.9) in those with mothers’ practice and 47.1 (95% CI: 42.0–52.8) in those with both parents’ practice.

**Table 2:** Determinant of students’ practice.

	No. of students	No. of students eating raw freshwater fish	Percentage (%)	OR (95% CI)	p	Adjusted OR (95% CI)^a^	p
**Genders**							
**Girls**	11679	1436	12.3				
**Boys**	11543	2282	19.8	1.8 (1.6–1.9)	0.000	2.1 (1.9–2.3)	0.000
**Ages**^**b**^	23222	3718	16.0	1.1 (1.1–1.1)	0.000	1.1 (1.1–1.1)	0.000
**Knowledge on transmission route**							
**No**	15216	2290	15.0				
**Yes**	8006	1428	17.8	1.2 (1.1–1.3)	0.000		
**Knowledge on early symptoms**							
**No**	16827	2466	14.7				
**Yes**	6395	1252	19.6	1.4 (1.3–1.5)	0.000	1.2 (1.1–1.3)	0.000
**Knowledge on complications**							
**No**	19358	3117	16.1				
**Yes**	3864	601	15.6	1.0 (0.9–1.1)	0.396		
**Knowledge on carcinogenicity**							
**No**	21445	3478	16.2				
**Yes**	1777	240	13.5	0.8 (0.7–0.9)	0.003		
**Parents' eating raw freshwater fish**					0.000		0.000
**Neither**	15929	566	3.6				
**Fathers eating**	3995	1099	27.5	10.3 (9.2–11.5)	0.000	10.5 (9.4–11.7)	0.000
**Mothers eating**	307	165	53.7	31.5 (24.8–40.1)	0.000	33.6 (26.3–42.9)	0.000
**Both**	2991	1888	63.1	46.5 (41.5–52.0)	0.000	47.1 (42.0–52.8)	0.000

^a^ Genders, ages, knowledge on early symptoms, and parents' practice were included in multivariate logistic regression.

^b^ Ages were a continuous variable in logistic regression analysis.

### Interaction of parents’ practice

Among the families with fathers eating raw freshwater fish, 42.8% of mothers ate raw fish, compared to 1.9% in those families without fathers eating (*χ*^2^ = 6713.1, p<0.001). When fathers ate raw freshwater fish, the RR for mothers’ raw-eating practice was 22.6 (95% CI: 20.0–25.6). Among the families with mothers eating, 90.7% of fathers ate raw fish, compared to 20.1% in those families without mothers eating. Thus, when mothers ate raw fish, the RR for fathers’ raw-eating practice was 4.5 (95% CI: 4.0–5.1).

### Belief and determinants in students

The percentage of students with the belief on the ability not to eat raw freshwater fish in future if required was lower in boys (77.6%) than in girls (86.2%) (*χ*^2^ = 287.6, p<0.001). Except in age 9, the difference was demonstrated significantly in all other ages (**S2 Table**). There showed significant linear decreasing trends by ages in both genders (**Table 1**). The corresponding *R*^2^ was 0.44, 0.81 and 0.70 in girls (p<0.05), boys (p<0.001) and both (p<0.01), respectively. Based on the linear trends, the belief decreased by 7.0% in girls and 15.8% in boys from 9 to 18. The annual decreasing rates were 0.8% and 1.9%, correspondingly.

After adjusted other factors, boys were less probable not to eat raw freshwater fish in future (aOR = 0.6, 95% CI: 0.5–0.6) (**Table 3**). Following the increase of age by one year, the aOR decreased to 0.9 (95% CI: 0.9–0.9). Compared to those without the knowledge on transmission route, the aOR was 1.4 (95% CI: 1.3–1.5) in those with the knowledge. If the students knew early symptoms, the aOR increased to 1.1 (95% CI: 1.1–1.2). Compared to those without the practice of eating raw freshwater fish, those students with the practice had an aOR of 0.6 (95% CI: 0.5–0.6). Compared to those without parents’ raw-eating practice, the aOR was 0.8 (95% CI: 0.7–0.9) in those with fathers’ practice, 0.7 (95% CI: 0.5–0.9) in those with mothers’ practice and 0.7 (95% CI: 0.6–0.8) in those with both parents’ practice.

**Table 3:** Determinant of students’ belief.

	No. of students	No. of students with the belief	Percentage (%)	OR (95% CI)	p	Adjusted OR (95% CI)^a^	p
**Genders**							
**Girls**	11679	10065	86.2				
**Boys**	11543	8959	77.6	0.6 (0.5–0.6)	0.000	0.6 (0.5–0.6)	0.000
**Ages**^**b**^	23222	19024	81.9	0.9 (0.9–0.9)	0.000	0.9 (0.9–0.9)	0.000
**Knowledge on transmission route**							
**No**	15216	12251	80.5				
**Yes**	8006	6773	84.6	1.3 (1.2–1.4)	0.000	1.4 (1.3–1.5)	0.000
**Knowledge on early symptoms**							
**No**	16827	13728	81.6				
**Yes**	6395	5296	82.8	1.1 (1.0–1.2)	0.029	1.1 (1.1–1.2)	0.001
**Knowledge on complications**							
**No**	19358	15817	81.7				
**Yes**	3864	3207	83.0	1.1 (1.0–1.2)	0.057		
**Knowledge on carcinogenicity**							
**No**	21445	17537	81.8				
**Yes**	1777	1487	83.7	1.1 (1.0–1.3)	0.045		
**Students' eating raw freshwater fish**							
**No**	19504	16456	84.4				
**Yes**	3718	2568	69.1	0.4 (0.4–0.4)	0.000	0.6 (0.5–0.6)	0.000
**Parents' eating raw freshwater fish**					0.000		0.000
**Neither**	15929	13490	84.7				
**Fathers eating**	3995	3145	78.7	0.7 (0.6–0.7)	0.000	0.8 (0.7–0.9)	0.000
**Mothers eating**	307	226	73.6	0.5 (0.4–0.7)	0.000	0.7 (0.5–0.9)	0.004
**Both**	2991	2163	72.3	0.5 (0.4–0.5)	0.000	0.7 (0.6–0.8)	0.000

^a^ Genders, ages, knowledge on transmission route, knowledge on early symptoms, students' practice and parents' practice were included in multivariate logistic regression.

^b^ Ages were a continuous variable in logistic regression analysis.

## Discussion

This study demonstrated the significant familial aggregation in raw-freshwater fish-eating practice indicated by the β-binomial distribution other than binomial distribution. Thus, familial assimilation functions in this dietary practice. The clustering coefficient increased by the ages of students, which indicates that familial assimilation strengthens following the extension of a family’s establishment. By further analysis on the practice between children and parents, as well as between spouses, it is demonstrated that familial assimilation consists of two components, namely intergenerational assimilation and martial assimilation. Intergenerational assimilation leads to the vertical transmission of raw-freshwater fish-eating practice from parents to their children. High effect indicated by the odd ratio was shown in the impact on students’ eating practice by their parents’ practice, especially when both parents did. Following the increase of students’ ages, raw-eating practice increased linearly, and the increase was higher in boys compared to girls. Furthermore, the clustering coefficient was higher in those families with boys compared to girls. Thus, intergenerational assimilation is characterized by the increasing effect with ages and higher effect in boys. Martial assimilation represents the horizontal transmission of raw-freshwater fish-eating practice in a family. Although husband and wife had contributions to counterpart’s raw-freshwater fish-eating practice, the effect is not equal, which demonstrates the differential interaction in martial assimilation. It was presented through the indicator of relative risk that husband has higher impact on his wife than that of wife on her husband. This difference indicates the dominance of husband in martial assimilation, which is consistent to the characteristics in intergenerational assimilation-higher effect in boys than girls. Thus, we argue that familial assimilation in transmission of raw-freshwater fish-eating practice determines the persistent high endemicity of clonorchiasis in China. Furthermore, the profiles of familial assimilation determine the epidemiology of clonorchiasis characterized by the increasing prevalence by ages and higher prevalence in males compared to females [1, 11, 13]. Thus, it is reasonable to propose that an individual gradually gets accustomed to eating raw freshwater fish because of the intergenerational assimilation by their parents and then promotes his/her spouse to do through martial assimilation when he/she is married. Then, the new spouses transmit the raw-freshwater fish-eating practice to a new generation, which leads to the persistent circulation of the raw-eating practice and subsequent clonorchiasis. The familial aggregation of *C*. *sinensis* infections in China and South Korea supports this hypothesis [11, 25]. Furthermore, it has also been demonstrated that in Laos *Opisthorchis viverrini* infection in mothers contributed to their children’s infection, although the infection status of fathers was not analyzed [26]. Especially, this hypothesis could explain the persistent endemicity of clonorchiasis in eastern China for over one century, as well as the persistent of clonorchiasis and opisthorchiasis in other Asian countries [8, 12].

The belief of students decreased by ages, and it was lower in boys compared to girls and impacted negatively by parents’ practice. This is reasonable, which showed reverse effects compared to the raw-eating practice because the belief inquired the ability not to do in future. The negative impact by the existing practice on the belief was also demonstrated, which was even higher than other factors indicated by a higher odds ratio. This implies that although students’ practice is highly influenced by their parents, the belief would be more determined by their existing practice. This just explains why adults have more knowledge but are more difficult to abandon their raw-eating practice through education [22]. In other words, behavioral intervention on raw-eating practice should be implemented at early stage.

What is difficult to understand is that more knowledge on the early symptoms was possessed when students ate raw freshwater fish. Eating raw freshwater fish could cause clonorchiasis, in which early symptoms including abdominal pain and diarrhoea usually presents [12]. Thus, those students with raw-eating practice would have more experiences in these symptoms, which led to the positive relevance of this piece of knowledge with raw-eating practice. However, this phenomenon was avoided in the belief. Because the belief inquired the future practice when the knowledge had already possessed. Thus, there exists clear time effect. It was demonstrated that such knowledge as the transmission route and early symptoms has protective effect in belief. However, the knowledge on complications and carcinogenicity has no effect. On the one hand, the knowledge on these conditions was lower. On the other hand, the complications and carcinogenicity could only be presented after long and chronic infection with *C*. *sinensis* [1, 12], and thus students usually have no subjective impression on these conditions. This study completely demonstrates the low knowledge on clonorchiasis in China, which has only been shown in local surveys before [22, 27]. Thus, education interventions should be employed through school platforms. The successful adoption of educational videos including cartoons in schistosomiasis and soil-transmitted helminthiases sets examples for clonorchiasis [28–31].

Thus, to control clonorchiasis sustainably, familial assimilation in transmission of raw-freshwater fish-eating practice should be interrupted, especially the vertical intergenerational assimilation from parents to children. When traditional chemotherapy benefits the output of infected population, the blocking of intergenerational assimilation will decrease the input of new cases. Thus, behavioral intervention through education should be integrated into the strategy against clonorchiasis. However, behavioral intervention should be implemented as early as possible especially before the establishment of raw-eating practice in the childhood. Additionally, the findings here might be also valuable to other food-borne diseases.

There exist several limitations in our study. Firstly, only qualitative information on ingestion of raw freshwater fish was collected. The capture of quantitative frequency will benefit further understanding in transmission mechanism. Additionally, although this study explored the practice in ingestion of raw freshwater fish, the types of diverse raw freshwater fishes ingested by habitants were not classified. Secondly, the raw-eating practice of parents was only indirectly collected from their children, which may be not very accurate and didn’t take consideration of the different impact of parents’ eating raw freshwater fish on their children in different occasions. Thirdly, infection status of *C*. *sinensis* was not determined in this study. These deserve to be explored in future studies, which will contribute to fully capture the determinants of clonorchiasis and then design intervention measures more effectively.

## Supporting information

S1 Checklist STROBE checklist. (PDF)

S1 Table Raw-freshwater fish-eating practice of students by genders and ages. (DOCX)

S2 Table Belief of students by genders and ages. (DOCX)
