# Supplementary material for: Familial assimilation in transmission of raw-freshwater fish-eating practice leading to clonorchiasis
Source: PLoS Negl Trop Dis. 2020 Apr 30;14(4):e0008263. doi: 10.1371/journal.pntd.0008263 (PMC7233597; doi:10.1371/journal.pntd.0008263)
Supplement: S1 Table — (DOCX) [file pntd.0008263.s002.docx]

## S1 Table. Raw-freshwater fish-eating practice of students by genders and ages

| **Ages** | **Genders** | **No** | **Yes** | **Number of students** | **Percentage of “Yes” (%)** | **χ^2^** | **p** |
| --- | --- | --- | --- | --- | --- | --- | --- |
| **9** | Girls | 368 | 34 | 402 | 8.46 | 4.501 | 0.034 |
|  | Boys | 330 | 50 | 380 | 13.16 |  |  |
|  | Subtotal | 698 | 84 | 782 | 10.74 |  |  |
| **10** | Girls | 998 | 98 | 1096 | 8.94 | 6.127 | 0.013 |
|  | Boys | 1004 | 139 | 1143 | 12.16 |  |  |
|  | Subtotal | 2002 | 237 | 2239 | 10.59 |  |  |
| **11** | Girls | 1312 | 143 | 1455 | 9.83 | 27.535 | 0.000 |
|  | Boys | 1276 | 249 | 1525 | 16.33 |  |  |
|  | Subtotal | 2588 | 392 | 2980 | 13.15 |  |  |
| **12** | Girls | 1315 | 172 | 1487 | 11.57 | 16.436 | 0.000 |
|  | Boys | 1357 | 271 | 1628 | 16.65 |  |  |
|  | Subtotal | 2672 | 443 | 3115 | 14.22 |  |  |
| **13** | Girls | 1595 | 235 | 1830 | 12.84 | 18.059 | 0.000 |
|  | Boys | 1424 | 312 | 1736 | 17.97 |  |  |
|  | Subtotal | 3019 | 547 | 3566 | 15.34 |  |  |
| **14** | Girls | 1513 | 212 | 1725 | 12.29 | 56.894 | 0.000 |
|  | Boys | 1298 | 367 | 1665 | 22.04 |  |  |
|  | Subtotal | 2811 | 579 | 3390 | 17.08 |  |  |
| **15** | Girls | 1315 | 182 | 1497 | 12.16 | 74.982 | 0.000 |
|  | Boys | 1110 | 359 | 1469 | 24.44 |  |  |
|  | Subtotal | 2425 | 541 | 2966 | 18.24 |  |  |
| **16** | Girls | 891 | 157 | 1048 | 14.98 | 36.932 | 0.000 |
|  | Boys | 674 | 237 | 911 | 26.02 |  |  |
|  | Subtotal | 1565 | 394 | 1959 | 20.11 |  |  |
| **17** | Girls | 616 | 137 | 753 | 18.19 | 15.259 | 0.000 |
|  | Boys | 482 | 177 | 659 | 26.86 |  |  |
|  | Subtotal | 1098 | 314 | 1412 | 22.24 |  |  |
| **18** | Girls | 320 | 66 | 386 | 17.10 | 14.459 | 0.000 |
|  | Boys | 306 | 121 | 427 | 28.34 |  |  |
|  | Subtotal | 626 | 187 | 813 | 23.00 |  |  |
| **Total** | Girls | 10243 | 1436 | 11679 | 12.30 | 241.154 | 0.000 |
|  | Boys | 9261 | 2282 | 11543 | 19.77 |  |  |
|  | Total | 19504 | 3718 | 23222 | 16.01 |  |  |
