# Supplementary material for: Familial assimilation in transmission of raw-freshwater fish-eating practice leading to clonorchiasis
Source: PLoS Negl Trop Dis. 2020 Apr 30;14(4):e0008263. doi: 10.1371/journal.pntd.0008263 (PMC7233597; doi:10.1371/journal.pntd.0008263)
Supplement: S2 Table — (DOCX) [file pntd.0008263.s003.docx]

## S2 Table. Belief of students by genders and ages

| **Ages** | **Genders** | **No/uncertain** | **Yes** | **Number of students** | **Percentage of “Yes” (%)** | **χ^2^** | **p** |
| --- | --- | --- | --- | --- | --- | --- | --- |
| **9** | Girls | 65 | 337 | 402 | 83.83 | 1.460 | 0.227 |
|  | Boys | 74 | 306 | 380 | 80.53 |  |  |
|  | Subtotal | 139 | 643 | 782 | 82.23 |  |  |
| **10** | Girls | 139 | 957 | 1096 | 87.32 | 24.455 | 0.000 |
|  | Boys | 234 | 909 | 1143 | 79.53 |  |  |
|  | Subtotal | 373 | 1866 | 2239 | 83.34 |  |  |
| **11** | Girls | 137 | 1318 | 1455 | 90.58 | 30.499 | 0.000 |
|  | Boys | 247 | 1278 | 1525 | 83.80 |  |  |
|  | Subtotal | 384 | 2596 | 2980 | 87.11 |  |  |
| **12** | Girls | 157 | 1330 | 1487 | 89.44 | 31.791 | 0.000 |
|  | Boys | 287 | 1341 | 1628 | 82.37 |  |  |
|  | Subtotal | 444 | 2671 | 3115 | 85.75 |  |  |
| **13** | Girls | 267 | 1563 | 1830 | 85.41 | 34.807 | 0.000 |
|  | Boys | 386 | 1350 | 1736 | 77.76 |  |  |
|  | Subtotal | 653 | 2913 | 3566 | 81.69 |  |  |
| **14** | Girls | 248 | 1477 | 1725 | 85.62 | 47.040 | 0.000 |
|  | Boys | 393 | 1272 | 1665 | 76.40 |  |  |
|  | Subtotal | 641 | 2749 | 3390 | 81.09 |  |  |
| **15** | Girls | 227 | 1270 | 1497 | 84.84 | 59.594 | 0.000 |
|  | Boys | 392 | 1077 | 1469 | 73.32 |  |  |
|  | Subtotal | 619 | 2347 | 2966 | 79.13 |  |  |
| **16** | Girls | 163 | 885 | 1048 | 84.45 | 39.352 | 0.000 |
|  | Boys | 247 | 664 | 911 | 72.89 |  |  |
|  | Subtotal | 410 | 1549 | 1959 | 79.07 |  |  |
| **17** | Girls | 134 | 619 | 753 | 82.20 | 29.324 | 0.000 |
|  | Boys | 198 | 461 | 659 | 69.95 |  |  |
|  | Subtotal | 332 | 1080 | 1412 | 76.49 |  |  |
| **18** | Girls | 77 | 309 | 386 | 80.05 | 9.890 | 0.002 |
|  | Boys | 126 | 301 | 427 | 70.49 |  |  |
|  | Subtotal | 203 | 610 | 813 | 75.03 |  |  |
| **Total** | Girls | 1614 | 10065 | 11679 | 86.18 | 287.644 | 0.000 |
|  | Boys | 2584 | 8959 | 11543 | 77.61 |  |  |
|  | Total | 4198 | 19024 | 23222 | 81.92 |  |  |
